# Andexanet alfa versus four-factor prothrombin complex concentrate for the reversal of apixaban- or rivaroxaban-associated intracranial hemorrhage: a propensity score-overlap weighted analysis

**DOI:** 10.1186/s13054-022-04043-8

**Published:** 2022-06-16

**Authors:** Olivia S. Costa, Stuart J. Connolly, Mukul Sharma, Jan Beyer-Westendorf, Mary J. Christoph, Belinda Lovelace, Craig I. Coleman

**Affiliations:** 1 Department of Pharmacy Practice, University of Connecticut School of Pharmacy, 69 North Eagleville Road, Unit 3092, Storrs, CT 06269 USA; 2 Evidence-Based Practice Center, Hartford Hospital, Hartford, CT USA; 3 Department of Medicine, McMaster University, 237 Barton St East, Hamilton, ON L8L 2X2 Canada; 4 Population Health Research Institute, Hamilton, ON Canada; 5 Thrombosis and Anticoagulation Service, Division Hematology and Hemostaseology, Department of Medicine I, Dresden University Hospital, Fetscherstrasse 74, 01307 Dresden, Germany; 6 Department of Global Health Economics and Outcomes Research, Alexion, AstraZeneca Rare Disease, 121 Seaport Blvd, Boston, MA 02210 USA

**Keywords:** Andexanet alfa, Four-factor prothrombin complex concentrate, Direct factor Xa inhibitor reversal, Intracranial hemorrhage

## Abstract

**Background:**

Andexanet alfa is approved (FDA “accelerated approval”; EMA “conditional approval”) as the first specific reversal agent for factor Xa (FXa) inhibitor-associated uncontrolled or life-threatening bleeding. Four-factor prothrombin complex concentrates (4F-PCC) are commonly used as an off-label, non-specific, factor replacement approach to manage FXa inhibitor-associated life-threatening bleeding. We evaluated the effectiveness and safety of andexanet alfa versus 4F-PCC for management of apixaban- or rivaroxaban-associated intracranial hemorrhage (ICH).

**Methods:**

This two-cohort comparison study included andexanet alfa patients enrolled at US hospitals from 4/2015 to 3/2020 in the prospective, single-arm ANNEXA-4 study and a synthetic control arm of 4F-PCC patients admitted within a US healthcare system from 12/2016 to 8/2020. Adults with radiographically confirmed ICH who took their last dose of apixaban or rivaroxaban < 24 h prior to the bleed were included. Patients with a Glasgow Coma Scale (GCS) score < 7, hematoma volume > 60 mL, or planned surgery within 12 h were excluded. Outcomes were hemostatic effectiveness from index to repeat scan, mortality within 30 days, and thrombotic events within five days. Odds ratios (ORs) with 95% confidence intervals (CI) were calculated using propensity score-overlap weighted logistic regression.

**Results:**

The study included 107 andexanet alfa (96.6% low dose) and 95 4F-PCC patients (79.3% receiving a 25 unit/kg dose). After propensity score-overlap weighting, mean age was 79 years, GCS was 14, time from initial scan to reversal initiation was 2.3 h, and time from reversal to repeat scan was 12.2 h in both arms. Atrial fibrillation was present in 86% of patients. Most ICHs were single compartment (78%), trauma-related (61%), and involved the intracerebral and/or intraventricular space(s) (53%). ICH size was ≥ 10 mL in volume (intracerebral and/or ventricular) or ≥ 10 mm in thickness (subdural or subarachnoid) in 22% of patients and infratentorial in 15%. Andexanet alfa was associated with greater odds of achieving hemostatic effectiveness (85.8% vs. 68.1%; OR 2.73; 95% CI 1.16–6.42) and decreased odds of mortality (7.9% vs. 19.6%; OR 0.36; 95% CI 0.13–0.98) versus 4F-PCC. Two thrombotic events occurred with andexanet alfa and none with 4F-PCC.

**Conclusions:**

In this indirect comparison of patients with an apixaban- or rivaroxaban-associated ICH, andexanet alfa was associated with better hemostatic effectiveness and improved survival compared to 4F-PCC.

*Trial registration* NCT02329327; registration date: December 31, 2014.

## Background

Since their approval by the US Food and Drug Administration (FDA) and the European Medicine Agency (EMA) in 2010, direct-acting oral anticoagulants (DOACs) and in particular the factor Xa (FXa) inhibitors apixaban and rivaroxaban have been increasingly utilized to treat or prevent various thrombotic disease states [1–4]. While DOACs are associated with a decreased risk of intracranial hemorrhage compared to vitamin K antagonists (VKAs), these life-threatening bleeds were observed in DOAC-treated patients at a rate of approximately 0.7%/year in randomized trials [5]. The previous literature has shown that antithrombotic-related intracranial hemorrhages can result in a high clinical burden, with in-hospital mortality rates of 12.4% for traumatic and 29% for non-traumatic intracranial hemorrhage [6]. Research in the American Heart Association’s Get with the Guidelines Stroke registry also found a high burden of in-hospital mortality of 27% for patients with spontaneous intracerebral hemorrhage in the presence of FXa inhibitors, with odds of mortality being significantly higher for patients taking FXa inhibitors compared to those who do not take anticoagulants, but significantly lower compared to those taking warfarin [7].

In 2018 and 2019 (respectively), the first specific FXa inhibitor reversal agent, coagulation FXa (recombinant), inactivated-zhzo (US adopted name: andexanet alfa), was approved through an accelerated pathway by FDA and conditionally approved by EMA for patients treated with rivaroxaban or apixaban when reversal of anticoagulation is needed for those experiencing life-threatening or uncontrolled bleeding [8, 9]. Andexanet alfa is a modified, recombinant, inactive form of human FXa developed to serve as a decoy to bind FXa inhibitor molecules and reduce anti-FXa activity [10]. In the single-arm prospective Andexanet Alfa, a Novel Antidote to the Anticoagulation Effects of Factor Xa Inhibitors (ANNEXA-4) study, treatment with andexanet resulted in a reduction of 92% in anti-FXa activity and 82% of patients were adjudicated as having effective hemostasis [11]. Factor concentrates, most notably four-factor prothrombin complex concentrate (4F-PCC), have been used as alternative off-label strategies for the management of major bleeding despite an absence of prospective clinical trial data [12, 13].

There is currently no randomized controlled trial evaluating the comparative effectiveness and safety of andexanet alfa and 4F-PCC for the management of severe or life-threatening bleeds. To aid in bridging this data gap, we conducted an indirect comparative study using ANNEXA-4 data and a synthetic control arm. Utilizing this approach, we sought to evaluate the effectiveness and safety of andexanet alfa versus 4F-PCC in the management of apixaban- or rivaroxaban-associated intracranial hemorrhage in a US patient population.

## Methods

### Study population

We evaluated patients who developed an intracranial hemorrhage while treated with apixaban or rivaroxaban and were managed with either andexanet alfa or 4F-PCC. This analysis was an indirect comparison utilizing data from a clinical trial and data from an observational study to serve as a synthetic control arm, since the clinical trial did not enroll control patients, whereas the observational study database did not contain data on andexanet alfa-treated patients. The clinical trial included 477 patients with acute major bleeding while receiving a FXa inhibitor enrolled between April 11, 2015, through March 30, 2020, in the multicenter, prospective, open-label, single-group ANNEXA-4 study (ClinicalTrials.gov Identifier: NCT02329327) [11]. The ANNEXA-4 design has been previously described [11]. For this analysis, only trial participants recruited in the USA were included to reduce systematic differences related to differing guidelines and clinical practices in bleed management across different countries between the two arms.

The synthetic control arm [14] used electronic health record (EHR) data for patients admitted between December 1, 2016, and August 30, 2020, to one of three acute care hospitals within Hartford Healthcare, a single healthcare system in the Northeastern USA.

### Inclusion and exclusion criteria

To be included in this analysis, patients in both arms had to be ≥ 18 years of age; to be admitted to a US hospital for a radiographically confirmed (computed tomography [CT] or magnetic resonance imaging [MRI]) acute intracranial hemorrhage defined as a spontaneous or traumatic bleed in the intracerebral, subdural, or subarachnoid space(s); and to have taken apixaban or rivaroxaban within 24 h of the bleed. Exclusion criteria for this analysis included a Glasgow Coma Scale (GCS) score of < 7 upon admission, an intracerebral bleed volume > 60 mL upon index CT/MRI scan, or planned surgery within 12 h of the index scan.

### Propensity score-overlap weighting

To adjust for potential confounding between the andexanet alfa and 4F-PCC arms, we calculated propensity scores based upon multivariable logistic regression [15], which included baseline age (continuous), sex (binary), body mass index (continuous), creatinine clearance (continuous), atrial fibrillation as the indication for oral anticoagulation (binary), average systolic blood pressures at admission and immediately prior to reversal administration > 160 mm Hg (binary), medical history of heart failure (binary), diabetes (binary), myocardial infarction (binary) or stroke (binary), concomitant antiplatelet use (binary), time from index scan to reversal agent initiation (continuous), time from end of reversal agent administration to repeat scan (continuous), traumatic versus spontaneous bleeding (binary), infratentorial region involvement (binary), single- versus multicompartment bleed (binary), bleeding in the intracerebral/intraventricular (binary), subdural (binary), or subarachnoid (binary) space on index scan, and bleed size ≥ 10 mL in volume (intracerebral and/or ventricular) or ≥ 10 mm in thickness (for subdural or subarachnoid) on index scan (binary). The area under the curve for resulting propensity scores predicting andexanet alfa versus 4F-PCC use was 0.82, 95% CI 0.75–0.88.

Estimated propensity scores were subsequently used to weight patients for analysis using an overlap weighting approach. Overlap weighting [16, 17] assigns weights to patients that are proportional to their probability of belonging to the opposing treatment cohort (i.e., andexanet alfa patients were weighted by the probability of receiving 4F-PCC (or 1 – the propensity score), and 4F-PCC patients were weighted by the probability of receiving andexanet alfa (the propensity score). Overlap weighting was chosen for confounder adjustment in this study because it allows for all eligible patients to be included in the analysis unlike propensity score matching, which typically results in sample size reduction in one or both cohorts. Overlap weighting assigns greater weight to patients in which treatment cannot be predicted and lesser weight to patients with extreme propensity scores (approaching 0.0 or 1.0). This prevents these outliers from dominating the analysis and decreasing precision (a concern when using inverse probability weighting). It also has the favorable property of resulting in the exact balance of all variables included in the logistic regression model used to derive the propensity scores [15–18].

### Outcomes

The co-primary outcomes for this study were hemostatic effectiveness (excellent/good vs. poor/none) and 30-day all-cause mortality. Excellent/good hemostasis was defined as ≤ 35% increase in hematoma size from index to repeat scan at approximately 12 h after reversal administration [11, 19]. The repeat scan closest to 12 h was utilized whenever possible. If no repeat scan was available within 12 ± 5 h of reversal administration, then the worst scan within 24 h was used. If no repeat scan was available within 24 h, the patient was assumed to have had poor hemostatic effectiveness. Patients whose index or a repeat scan could not be accessed due to administrative reasons were excluded from this study (andexanet alfa: *n* = 7, 4F-PCC: *n* = 5). Volume was calculated for intracerebral and intraventricular bleeds using the abc/2 method [20] while thickness was measured for subarachnoid and subdural hemorrhages.

Adjudicated hemostatic efficacy determinations from ANNEXA-4 [11] were used for all andexanet alfa patients included in this study. A similar process was used to determine hemostatic effectiveness for patients treated with 4F-PCC, where an adjudication committee determined hemostatic efficacy in patients with ambiguous outcomes. Patients receiving 4F-PCC had their index and repeat scans read by two independent investigators. Hemostatic effectiveness for 4F-PCC patients was then adjudicated by investigator consensus using the published ANNEXA-4 criteria [11]. If a patient had a multicompartmental bleed with contradictory hematoma size change, hemostatic efficacy was determined by achieving consensus between two independent investigators.

Secondary outcomes included thrombotic event occurrence during the first five days after reversal agent administration. The five-day time frame for thrombotic events was selected as it was a time point specifically reported in ANNEXA-4 and it reduced potential surveillance bias (which can occur when outcomes are sought with differential intensity across populations or over time, or according to care setting and/or patient characteristics) associated with post-discharge thrombotic events [6, 11].

### Statistical analysis

Baseline characteristics were analyzed using descriptive statistics. Categorical variables were reported as percentage and continuous variables as means ± standard deviations. Propensity score model–eligible variables with < 10% missing data had missing values imputed using a multiple imputation approach based on a fully conditional specification linear regression model including all available covariates and outcomes [21]. Absolute standardized differences (ASDs) were calculated for each variable prior to propensity score-overlap weighting to illustrate the magnitude of imbalance between arms at baseline (an ASD > 0.1 was considered to represent a relevant difference) [15].

For hemostatic effectiveness, mortality, and thrombotic events, propensity score-overlap weighted binomial (logit) generalized estimating equations with a robust estimator were used to calculate odds ratios (ORs) with accompanying 95% confidence intervals (CIs) [17].

We performed a sensitivity analysis whereby attainment of hemostatic effectiveness (or lack thereof) for patients without a repeat scan within 24 h was adjudicated based on clinical judgement by two independent investigators after a review of outcomes (e.g., need for unplanned surgery, subsequent administration of reversal agent, mortality) rather than being assumed to represent poor/no effectiveness. Propensity scores were re-calculated for the sensitivity analysis.

A subgroup analysis of patients with only an intracerebral and/or intraventricular hemorrhage and available pre- and post-scans evaluated the above-mentioned outcomes and the absolute change in hematoma volume in mL from index to repeat scan. The subgroup analysis used propensity scores based on a logistic regression model where total bleed volume in mL on index scan (continuous) was substituted for bleed size ≥ 10 mL/mm. A propensity score-overlap weighted linear generalized estimating equation with a robust estimator was used to determine the mean difference between andexanet alfa and 4F-PCC in hematoma volume from index to repeat scan.

In all cases, a *P* value < 0.05 was considered statistically significant. All database management and statistical analysis were performed using IBM SPSS version 27.0 (IBM Corp., Armonk, NY). This report was written to comply with the Reporting of Studies Conducted Using Observational Routinely Collected Health Data for Pharmacoepidemiology (RECORD-PE) statement [22].

## Results

### Baseline characteristics and propensity score-overlap weighting

**Fig. 1:**
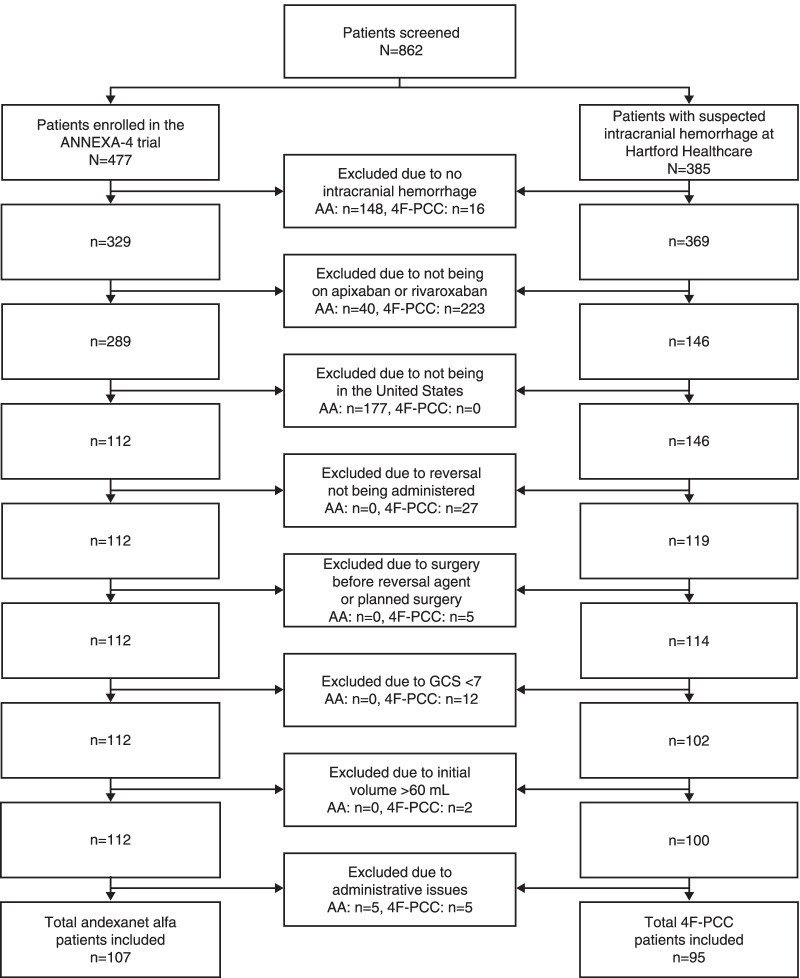
4F-PCC and AA patient identification. AA = andexanet alfa, 4F-PCC = four-factor prothrombin complex concentrate, GCS = Glasgow Coma Scale score

A total of 107 andexanet alfa patients enrolled at US sites from April 2015 through March 2020, and 95 4F-PCC patients admitted between December 2016 and August 2020 for intracranial hemorrhage were included in this analysis. Selection of andexanet alfa patients from ANNEXA-4 and 4F-PCC patients for the synthetic control arm using inclusion and exclusion criteria is detailed in Fig. 1.

Baseline characteristics prior to propensity score-overlap weighting are reported in Table 1. There were relevant differences in baseline characteristics of patients receiving andexanet alfa and 4F-PCC as evidenced by most covariates having an ASD > 0.1. Andexanet alfa patients had poorer renal function at baseline (mean creatinine clearance of 64 ± 28 mL/min) compared to 4F-PCC patients (mean creatinine clearance of 73 ± 44 mL/min), and a higher proportion were on concomitant antiplatelet therapy (33.6%) compared with 4F-PCC patients (24.2%). The time between end of reversal administration to repeat scan was longer for andexanet alfa (12.4 ± 1.1 h) than 4F-PCC (8.1 ± 5.1 h). A greater percentage of andexanet patients had a bleed size ≥ 10 mL/mm (33.6%) than 4F-PCC patients (14.7%). Bleeds were more frequently located in intracerebral and/or intraventricular or infratentorial locations in andexanet alfa patients compared to 4F-PCC patients (59.8% vs 48.4% and 16.8% vs 12.6%, respectively). However, more patients treated with 4F-PCC had systolic blood pressure > 160 mm Hg (20.0%) than patients treated with andexanet alfa (12.1%).

**Table 1:** Baseline characteristics before propensity score-overlap weighting

Variable	Andexanet alfa *n* = 107	4F-PCC *n* = 95	Absolute standardized difference
*Demographics*			
Age (years), mean ± SD	79 ± 8	77 ± 11	0.21
Male, %	49.5	52.6	0.07
Body mass index (kg/m^2^), mean ± SD	27 ± 7	28 ± 6	0.14
Creatinine clearance (mL/min), mean ± SD	64 ± 28	73 ± 44	0.24
Systolic blood pressure > 160 mm Hg, %^a^	12.1	20.0	0.33
GCS score^b^	14 ± 1	14 ± 2	0.00
*Anticoagulant indication and medical history, %*			
Anticoagulant indication, atrial fibrillation	87.9	82.1	0.25
Medical history of heart failure	18.7	23.2	0.15
Medical history of diabetes	28.0	27.4	0.02
Medical history of myocardial infarction	12.1	8.4	0.22
Medical history of stroke	21.5	24.2	0.08
Concomitant use of an antiplatelet	33.6	24.2	0.25
*Intracranial hemorrhage characteristics*			
Initial imaging to reversal start (hours), mean ± SD	2.6 ± 1.8	2.1 ± 1.9	0.30
End of reversal to repeat imaging (hours), mean ± SD	12.4 ± 1.1	8.1 ± 5.1	1.21
Traumatic onset, %	53.3	64.2	0.25
Infratentorial location, %	16.8	12.6	0.19
Size of bleed ≥ 10 mL/mm, %	33.6	14.7	0.59
Single compartment bleed, %	77.6	85.3	0.28
Intracerebral and/or intraventricular bleed, %^c^	59.8	48.4	0.25
Subdural bleed, %^c^	32.7	40.0	0.17
Subarachnoid bleed, %^c^	31.8	27.4	0.12
*Reversal agent dosing, %*^b^			
Andexanet alfa			
400 mg bolus + 440 mg infusion	96.3	–	–
800 mg bolus + 860 mg infusion	3.7	–	–
4F-PCC			
25 units/kg infusion^d^	–	74.3	–
50 units/kg infusion^d^	–	25.3	–

4F-PCC = four-factor prothrombin complex concentrate, GCS = Glasgow Coma Scale, IQR = interquartile range, SD = standard deviation

^a^Blood pressure reported was an average of measurements upon arrival and immediately prior to reversal agent administration for both cohorts

^b^Not included in the propensity score model due to lack of heterogeneity between groups at baseline

^c^Intracranial hemorrhage types add up to > 100% given a portion of patients had multicompartment bleeds

^d^The median (IQR) dose was 2028 units (1728–2393) for patients receiving 25 units/kg and 3443 units (2911–4208) for those receiving 50 units/kg

Following propensity score-overlap weighting, the two reversal agent arms were identical for all recorded covariates as intended by the methods (Table 2). Mean age was 79 years, creatinine clearance was 67 mL/min, body mass index was 28 kg/m^2^, and GCS score was 14. Mean time from initial scan to reversal initiation was 2.3 h and time from reversal agent administration to repeat scan was 12.2 h. Atrial fibrillation was the indication for oral anticoagulation in 86.4% of patients. Heart failure, diabetes, and prior stroke were comorbid conditions in > 20% of included patients. Most bleeds were a result of trauma (61.1%) and were present in only a single compartment (78.3%) on the index scan, with about 50% being intracerebral and/or intraventricular hemorrhages. Both reversal agents were administered at their lower dose in most patients. (96.6% of patients received 400 mg bolus + 440 mg infusion of andexanet alfa; 79.3% of patients received a 25 units/kg infusion of 4F-PCC.)

**Table 2:** Baseline characteristics after propensity score-overlap weighting

Variable	Andexanet alfa *n* = 107	4F-PCC *n* = 95
*Demographics*		
Age (years), mean ± SD	79 ± 8	79 ± 11
Male, %	49.6	49.6
Body mass index (kg/m^2^), mean ± SD	28 ± 7	28 ± 6
Creatinine clearance (mL/min), mean ± SD	67 ± 30	67 ± 39
Systolic blood pressure > 160 mm Hg, %^a^	18.3	18.3
GCS score, %^b^	14 ± 1	14 ± 2
*Anticoagulant indication and medical history, %*		
Anticoagulant indication, atrial fibrillation	86.4	86.4
Medical history of heart failure	22.3	22.3
Medical history of diabetes	28.7	28.7
Medical history of myocardial infarction	9.4	9.4
Medical history of stroke	24.0	24.0
Concomitant use of an antiplatelet	24.2	24.2
*Intracranial hemorrhage characteristics*		
Initial imaging to reversal start (hours), mean ± SD	2.3 ± 1.6	2.3 ± 2.1
End of reversal to repeat imaging (hours), mean ± SD	12.2 ± 1.1	12.2 ± 4.6
Traumatic onset, %	61.1	61.1
Infratentorial location, %	14.7	14.7
Size of bleed ≥ 10 mL/mm, %	21.7	21.7
Single compartment bleed, %	78.3	78.3
Intracerebral and/or intraventricular bleed, %^c^	53.3	53.3
Subdural bleed, %^c^	38.4	38.4
Subarachnoid bleed, %^c^	34.3	34.3
*Reversal agent dosing, %*^b^		
Andexanet alfa		
400 mg bolus + 440 mg infusion	96.6	–
800 mg bolus + 860 mg infusion	3.4	–
4F-PCC		
25 units/kg infusion	–	79.3
50 units/kg infusion	–	20.7

4F-PCC = four-factor prothrombin complex concentrate, GCS = Glasgow Coma Scale, SD = standard deviation

^a^Blood pressure reported was an average of measurements upon arrival and immediately prior to reversal agent administration

^b^Not included in the propensity score model

^c^Intracranial hemorrhage types add up to > 100% given a portion of patients had multicompartment bleeds

### Hemostatic effectiveness

**Fig. 2:**
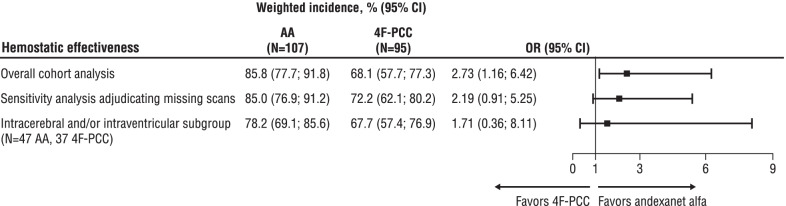
Odds of hemostatic effectiveness after propensity score-overlap weighting for andexanet alfa versus 4F-PCC (referent). AA = andexanet alfa, CI = confidence interval, 4F-PCC = four-factor prothrombin complex concentrate, OR = odds ratio

After propensity score-overlap weighting, the incidence of excellent/good hemostatic effectiveness was 85.8% for andexanet alfa compared to 68.1% for 4F-PCC (Fig. 2), corresponding to a propensity score-overlap weighted OR of 2.73 (95% CI 1.16–6.42) for andexanet alfa compared to 4F-PCC in achieving excellent/good hemostatic effectiveness in the overall cohort analysis. Two 4F-PCC patients with minimal change in index hematoma size upon follow-up were deemed to have poor/no hemostatic effectiveness due to the development of a new intraventricular and/or intracerebral hemorrhage upon repeat scan.

No significant differences in the hemostatic effectiveness results were shown in the sensitivity analysis, whereby patients without a repeat scan within 24 h had their attainment of hemostatic effectiveness adjudicated based on clinical judgement. Of the 95 4F-PCC patients, eight (8.4%) did not have a repeat scan (no andexanet alfa patients were impacted as we used the adjudicated hemostatic effectiveness determinations from ANNEXA-4). Four of these eight patients were deemed clinically stable after adjudication by two independent investigators, and their hemostatic effectiveness classification was changed from poor/none to excellent/good. Of the remaining four 4F-PCC patients, two required unplanned surgery, one was subsequently transferred to hospice care, and one died prior to hospital discharge. These four patients maintained their poor/no hemostatic effectiveness classification.

Upon subgroup analysis restricted to patients with a single compartment, intracerebral, and/or intraventricular hemorrhage, 47 andexanet alfa and 37 4F-PCC patients were available for analysis. Baseline characteristics of these patients after propensity score (re-calculated for the subgroup)–overlap weighting are provided in Table 3. Hemostatic effectiveness results were similar in direction and magnitude to those observed in the full analysis population with 78.2% (95% CI 69.1–85.6) of the andexanet alfa-treated and 67.7% (95% CI 57.4–76.9) of the 4F-PCC-treated population achieving hemostatic effectiveness, though differences in hemostatic effectiveness were not statistically significant in this subgroup analysis (OR 1.71 [95% CI 0.36–8.11] for achieving hemostatic effectiveness for andexanet alfa vs. 4F-PCC). Baseline total intracerebral and/or intraventricular hematoma volume was 7.3 mL in both arms. At the repeat scan, the increase in hematoma volume was 0.83 mL with andexanet alfa and 4.73 mL with 4F-PCC, a difference that was not statistically significant (Table 4).

**Table 3:** Baseline characteristics of the intracerebral and/or intraventricular subpopulation after propensity score-overlap weighting

Variable	Andexanet alfa *n* = 47	4F-PCC *n* = 37
*Demographics*		
Age (years), mean ± SD	77 ± 9	77 ± 10
Male, %	51.4	51.4
Body mass index (kg/m^2^), mean ± SD	28 ± 9	28 ± 6
Creatinine clearance (mL/min), mean ± SD	73 ± 32	73 ± 48
Systolic blood pressure > 160 mm Hg, %^a^	15.3	15.3
GCS score, %^b^	14 ± 1	13 ± 2
*Anticoagulant indication and medical history, %*		
Anticoagulation indication, atrial fibrillation	94.1	94.1
Medical history of heart failure	22.3	22.3
Medical history of diabetes	11.6	11.6
Medical history of myocardial infarction	10.7	10.7
Medical history of stroke	17.3	17.3
Concomitant use of an antiplatelet	22.7	22.7
*Intracranial hemorrhage characteristics*		
Initial image to reversal start (hours), mean ± SD	2.4 ± 1.9	2.4 ± 2.7
End of reversal to repeat image (hours), mean ± SD	12.1 ± 1.2	12.1 ± 5.0
Traumatic onset, %	30.8	30.8
Infratentorial location, %	23.3	23.3
Initial intracerebral and/or intraventricular volume (mL), mean ± SD	7.3 ± 9.8	7.3 ± 9.1
*Reversal agent dosing, %*^b^		
Andexanet alfa		
400 mg bolus + 440 mg infusion	95.2	–
800 mg bolus + 860 mg infusion	4.8	–
4F-PCC		
25 units/kg infusion	–	86.1
50 units/kg infusion	–	13.9

4F-PCC = four-factor prothrombin complex concentrate, GCS = Glasgow Coma Scale, SD = standard deviation

^a^Blood pressure reported was an average of the measurement at arrival and measurement just before reversal was administered

^b^Not included in propensity score

**Table 4:** Volume change between initial and repeat scan for intracerebral and/or intraventricular bleed subpopulation after propensity score-overlap weighting

	Andexanet alfa *n* = 47	4F-PCC *n* = 37
Initial volume (mL), mean ± SD	7.29 ± 9.82	7.29 ± 9.05
Repeat volume (mL), mean ± SD	8.12 ± 12.28	12.02 ± 16.82
Change in volume (mL), mean ± SD	0.83 ± 4.25	4.73 ± 12.10
Weighted difference in mean volume change (mL), 4F-PCC referent (95% CI)	− 3.90 (− 10.81 to 3.00)	

CI = confidence interval, 4F-PCC = four-factor prothrombin complex concentrate, SD = standard deviation

### 30-day mortality and thromboembolic events

**Fig. 3:**
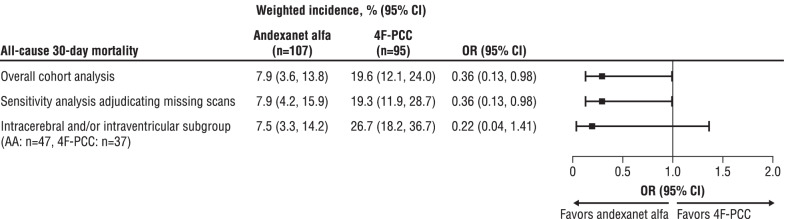
Odds of all-cause 30-day mortality after propensity score-overlap weighting for andexanet alfa versus 4F-PCC (referent). AA = andexanet alfa, CI = confidence interval, 4F-PCC = four-factor prothrombin complex concentrate, OR = odds ratio

Prior to propensity score-overlap weighting, 24 patients died during follow-up. Of the 10 andexanet alfa patients who died within 30-days, two (20%) were due to worsening intracranial hemorrhage, whereas seven of 14 (50%) patients administered 4F-PCC died due to worsening intracranial hemorrhage. Weighted incidence of 30-day all-cause mortality was 7.9% (95% CI 3.6–13.8) for patients in the andexanet alfa arm and 19.6% (95% CI 12.1–24.0) among patients in the 4F-PCC arm (Fig. 3) in the overall cohort analysis (weighted OR 0.36; 95% CI 0.13–0.98). The weighted incidence and weighted ORs for 30-day mortality were not impacted by the sensitivity analysis, wherein patients without a repeat scan within 24 h had their hemostatic effectiveness adjudicated based on clinical judgement. In the subgroup analysis of patients with intracerebral and/or intraventricular hemorrhage, weighted incidence of 30-day mortality rates was similar to the overall cohort analysis, although differences across groups were not statistically significant (7.5% [95% CI 3.3–14.2] for andexanet alfa and 26.7% [95% CI 18.2–36.7] for 4F-PCC; weighted OR 0.22 [95% CI 0.04–1.41]).

Regardless of andexanet alfa or 4F-PCC use, 30-day mortality was found to be common among patients with a baseline GCS score ≤ 12 (9/21 [42.9%] vs. 15/181 [4.3%], *p* < 0.001), infratentorial bleeding (7/30 [23.3%] vs. 17/172 [9.9%], *p* = 0.04), hematoma volume ≥ 10 mL/mm (11/50 [22.0%] vs. 13/152 [8.6%], *p* = 0.01) and multicompartment bleeding (9/38 [23.7%] vs. 15/164 [9.1%], *p* = 0.01).

Thrombotic events within five days of reversal agent administration were 2 for andexanet alfa and n = 0 for 4F-PCC for the overall cohort and sensitivity analysis. In the subgroup analysis including patients with intracerebral and/or intraventricular hemorrhage, there were no thromboembolic events within five days of reversal agent administration for either treatment arm.

## Discussion

The present study included > 200 patients experiencing an apixaban- or rivaroxaban-associated intracranial hemorrhage managed with either a target-specific (andexanet alfa) or nonspecific (4F-PCC) factor replacement agent. We found that treatment with andexanet alfa was associated with 2.7-fold higher odds of achieving hemostatic effectiveness and a 64% reduced relative odds of 30-day all-cause mortality compared to 4F-PCC. Thrombotic event incidence was not significantly different between groups. In a sensitivity analysis wherein 4F-PCC patients without repeat scans within 24 h were adjudicated rather than recorded as missing/poor, similar hemostatic effectiveness results were observed. A subgroup analysis restricted to single-compartment, intracerebral, and/or intraventricular hemorrhage patients demonstrated similar results to the full analysis population, along with an approximately 4 mL reduction in hematoma volume with andexanet alfa compared to 4F-PCC. Although the volume change between andexanet alfa versus 4F-PCC was nonsignificant in this subgroup analysis, it is possible that the small sample size was insufficient to detect differences across treatment arms.

The results of our analysis are generally consistent with prior, considerably smaller, comparative case series [23–25]. Barra and colleagues [26] reported a case series of apixaban- or rivaroxaban-associated intracranial hemorrhage patients managed with andexanet alfa (n = 18) or 4F-PCC (n = 11). The investigators reported that 89% of andexanet alfa and 60% of 4F-PCC patients achieved hemostatic effectiveness (defined as a ≤ 35% increase in subarachnoid or subdural hematoma thickness, or in intracerebral bleed volume). In-hospital mortality occurred in 22.2% of andexanet alfa and 63.6% of 4F-PCC patients, and thrombotic events occurred in 17% of andexanet alfa and 9% of 4F-PCC patients at 30 days. In another case series, Vestal and colleagues [23] reported on apixaban- or rivaroxaban-associated intracranial hemorrhage patients managed with andexanet alfa (n = 21) or 4F-PCC (n = 35). The investigators reported that 64.7% of andexanet alfa and 54.8% of 4F-PCC patients achieved hemostatic effectiveness (per radiologists’ interpretation). In-hospital mortality occurred in 14.3% of andexanet alfa and 37.1% of 4F-PCC patients, and thrombotic events occurred in 14.3% of andexanet alfa and 31.4% of 4F-PCC patients at 30 days. The investigators did not report data on 30-day all-cause mortality. Due to the report’s small sample size and substantial differences in patient and intracranial hemorrhage characteristics at baseline, no direct head-to-head comparison of the reversal strategies was performed. Moreover, the inclusion period for 4F-PCC patients started five years earlier than for andexanet alfa patients (July 2013–September 2019 compared to July 2018–September 2019), which may have introduced selection biases in results if comparison of reversal cohorts was attempted.

The results of this analysis also align with other indirect comparison studies for andexanet alfa versus usual care-treated patients with FXa inhibitor bleeding in the UK and Germany. Cohen and colleagues compared propensity score (model based on age, bleed location, history of atrial fibrillation, venous thromboembolism, stroke, renal dysfunction, and cancer)–adjusted cohorts of andexanet alfa- and PCC-managed FXa inhibitor major bleeding patients [24]. As in our study, the andexanet alfa cohort was derived from ANNEXA-4, while a synthetic control arm of PCC patients was drawn from the prospective, observational ORANGE study (which assessed the presentation and clinical outcomes of major bleeding episodes associated with oral anticoagulant use in the UK between October 2013 and August 2016) [25]. Among patients presenting with intracranial hemorrhage from either study (total n = 258), andexanet alfa use was associated with a 67% relative risk (RR) reduction in 30-day mortality (*p* < 0.001). Cohen and colleagues did not report data on hemostatic effectiveness or thrombotic outcomes. Huttner et al. compared an andexanet alfa cohort from ANNEXA-4 to a synthetic control arm of patients treated with “usual care” in patients with FXa inhibitor–related intracerebral hemorrhage derived from the German RETRACE II study [27]. Like our study, their analysis found a lower risk of poor/none hemostasis with andexanet alfa versus usual care (unadjusted incidences: 14% vs. 36%; adjusted RR = 0.40; 95% CI 0.20–0.78), however, there was no statistically significant difference in in-hospital mortality between the two treatments (unadjusted incidence: 16.5% vs. 20.6%; adjusted HR 0.49; 95% CI 0.24–1.04) [27]. The present analysis builds on these indirect comparisons by measuring outcomes within a large population of patients with intracranial hemorrhage within the USA.

Various international/national medical societies have developed guidelines regarding how to best manage FXa inhibitor-associated intracranial hemorrhage [28–37]. Current guidance from the American Heart Association/American College of Cardiology/Heart Rhythm Society [28], European Society of Cardiology [29], American College of Emergency Physicians [35], American Society of Hematology [30], European Stroke Organisation [31], and the German Society of Neurology [36] each recommends the use of andexanet alfa for the management of apixaban- or rivaroxaban-related severe life-threatening bleeding, with some [29, 31], but not all, recommending 4F-PCC use when andexanet alfa is unavailable or for non-life-threatening bleeds only. Guidance from other organizations, such as the International Society on Thrombosis and Haemostasis, Neurocritical Care Society, and American Heart Association/American Stroke Association, for spontaneous intracerebral hemorrhages were last updated prior to the approval of andexanet alfa and therefore cannot provide guidance on reversal agent selection [32–34]. The availability of real-world data reporting on the effectiveness and safety of andexanet alfa and 4F-PCC, including studies using ANNEXA-4 data with a synthetic control arm, may be helpful in shaping future clinical trials and eventually informing future guidelines.

### Study strengths and limitations

It is noteworthy that the present study is the largest comparison to date to assess the comparative effectiveness of andexanet alfa and 4F-PCC in the management of apixaban- or rivaroxaban-associated intracranial hemorrhage. Prior studies have compared andexanet alfa to any prothrombin complex concentrate [24] or usual care [27], whereas all patients in our study received 4F-PCC. The substantially overlapping time frame between the two arms in our study likely attenuated selection and chronologic biases present in earlier comparative studies. To reduce biases, data collected and outcome definitions used in the 4F-PCC arm were designed to mimic that of ANNEXA-4 whenever possible. Propensity scores were applied to reduce confounding by indication. The propensity score-overlap weighting allowed inclusion of all the patients in the dataset fulfilling inclusion/exclusion criteria in contrast to propensity score matching [15–17]. All patients in this study’s 4F-PCC arm had health insurance coverage (85% were Medicare patients), which should decrease the risk of socioeconomic factors influencing reversal agent choice in the synthetic control arm. Finally, to our knowledge, this study was the first large propensity score weighted study to compare andexanet alfa and 4F-PCC on three relevant clinical and safety outcomes: hemostatic effectiveness, mortality, and thrombotic complications.

Despite these strengths, our study has some limitations. First, we did not have data on incidence of early (within 24 h) do not resuscitate orders (DNR) or withdrawal of life-sustaining therapy. Patients receiving andexanet alfa and 4F-PCC were well-balanced on identified predictors of mortality (including baseline GCS score, infratentorial bleeding, larger hematoma volumes, and multicompartment bleeding) making a difference in the incidence of DNR orders and withdrawal of life-sustaining therapy between groups less likely. Second, we only reported thromboembolic events occurring in the first five days after reversal administration. While ANNEXA-4 [11] prospectively followed andexanet alfa patients for thrombotic events through 30 days, our dependence on EHR data (and not scheduled follow-up) for 4F-PCC patients made identification and verification of thrombotic events after 4F-PCC administration post-hospital discharge less reliable. Moreover, investigators in the prospective ANNEXA-4 study actively surveilled for thrombotic events, whereas clinicians in routine practice were less likely to do so, which could have biased the detection of thrombotic events against andexanet alfa. Of note, the five-day time point for thrombotic events utilized in our study was based on the time stratifications reported within the ANNEXA-4 study (< 6, 6–14, and 15–30 days after andexanet alfa administration) [11] and a recent study by Miao and colleagues [6], which demonstrated that five to six days is the median hospital length of stay for atrial fibrillation patients experiencing an intracranial hemorrhage. Third, based upon our study’s inclusion criteria, our findings are most applicable to an apixaban- or rivaroxaban-associated intracranial hemorrhage population with baseline GCS scores ≥ 8 and hematoma volumes ≤ 60 mL managed in the USA. Even though ANNEXA-4 included patients from the USA, Canada, and Europe [11], our analysis restricted inclusion in both arms to intracranial hemorrhage patients treated at a US hospital. This was deemed necessary to avoid potential confounding due to discrepancies in treatment practices from country to country (e.g., reversal agent dosing, time of repeat scans, etc.). Since 100% of 4F-PCC patients for this study were treated in the USA, “country” could not be included in our propensity score model (and weighted for). Additionally, the external validity of our results should be viewed in context of 4F-PCC dosing utilized in our study (which was at prescribers’ discretion). Approximately three out of every four patients in the 4F-PCC cohort of our study received a 25 u/kg dose of 4F-PCC. Some guidelines [31, 33] have recommended higher doses of 4F-PCC (37.5–50 u/kg) to reverse FXa inhibitor-associated uncontrolled or life-threatening bleeding, while others have endorsed low, fixed doses of 2,000 units [35, 37] or doses ranging between 10 and 25 units/kg with a repeated dose in 1 to 2 h if needed [35]. The level of evidence supporting these 4F-PCC dosing recommendations are generally acknowledged as low (based on studies assessing the correction of anticoagulant-induced laboratory abnormalities or punch biopsy studies) and the strength of recommendations deemed weak [31, 33, 35, 37]. Multiple studies comparing the hemostatic effectiveness of lower (25 u/kg, < 30 u/kg, or < 35 u/kg) and higher (≥ 30 u/kg, ≥ 35 u/kg, or 50 u/kg) 4F-PCC doses have failed to show significant differences between dosing strategies [38–40]. Fourth, as this was not a randomized controlled trial, the risk of confounding bias exists [38]. We attempted to mitigate this risk by implementing propensity score-overlap weighting [16, 17]; however, residual confounding due to unobserved or unmeasured covariates cannot be ruled out [14, 41]. Fifth, anti-FXa assays were not available in most 4F-PCC patients in the synthetic control arm since obtaining these levels was not standard practice. As a result, anti-FXa levels could not be adjusted for or used to assess effectiveness in the present study, and it is not known if patients in the 4F-PCC arm had similar levels of anticoagulation to those in the ANNEXA-4 trial. Next, our 4F-PCC arm included three patients with moderate to severe dementia. Due to the need to provide informed consent by patients or their medical proxies (unless an exception for informed consent for emergency procedures was obtained), such patients may have been excluded in ANNEXA-4. It is unclear what impact the inclusion of moderate to severe dementia patients may have had on our study’s results. Finally, due to insufficient sample size, our ability to perform subgroup and sensitivity analyses was limited. We performed a sensitivity analysis limited to a single compartment, intracerebral/intraventricular bleeds only. While this subanalysis was likely underpowered, the results were directionally consistent with the overall population analysis.

## Conclusions

Our indirect comparison analysis of ANNEXA-4-derived andexanet alfa patients and a synthetic control arm of 4F-PCC patients (~ 80% at 25 units/kg) from a US healthcare system showed that andexanet alfa was associated with better hemostatic effectiveness and reduced odds of all-cause mortality at 30 days. Our findings support current consensus guidelines published by international/national medical societies, which preferentially recommend the use of andexanet alfa over 4F-PCC for the management of apixaban- or rivaroxaban-associated life-threatening bleeds (including intracranial hemorrhage) [28–30]. Randomized controlled trials, such as the ANNEXA-I trial (ClinicalTrials.gov Identifier: NCT03661528), are ongoing in large and diverse populations comparing andexanet alfa to usual care.

## Data Availability

Alexion, AstraZeneca Rare Disease will consider requests for disclosure of clinical study participant-level data provided that participant privacy is assured through methods like data de-identification, pseudonymization, or anonymization (as required by applicable law), and if such disclosure was included in the relevant study informed consent form or similar documentation. Qualified academic investigators may request participant-level clinical data and supporting documents (statistical analysis plan and protocol) pertaining to Alexion-sponsored studies. Further details regarding data availability and instructions for requesting information are available in the Alexion Clinical Trials Disclosure and Transparency Policy at https://alexion.com/our-research/research-and-development. Link to Data Request Form (https://alexion.com/contact-alexion/medical-information).

## Abbreviations

ASD: Absolute standardized difference
CI: Confidence interval
CT: Computed tomography
DOAC: Direct-acting oral anticoagulant
EHR: Electronic health record
EMA: European Medicine Agency
FDA: Food and Drug Administration
4F-PCC: Four-factor prothrombin complex concentrate
GCS: Glasgow Coma Scale
MRI: Magnetic resonance imaging
OR: Odds ratio
RR: Relative risk
VKA: Vitamin K antagonist
